# Hypertriglyceridemia-Induced Pancreatitis: Choice of Treatment

**DOI:** 10.14740/gr662e

**Published:** 2015-07-22

**Authors:** Rafay Khan, Waqas Jehangir, Kalyani Regeti, Abdalla Yousif

**Affiliations:** a Department of Internal Medicine, Raritan Bay Medical Center, 530 New Brunswick Ave., Perth Amboy, NJ, USA

**Keywords:** Acute pancreatitis, Hypertriglyceridemia, Apheresis, Insulin

## Abstract

Severe hypertriglyceridemia is one of the many yet rare risk factors associated with acute pancreatitis. The level of triglycerides plays a crucial role in determining the method and duration of treatment. As with the treatment of other causes of acute pancreatitis, bowel rest, intravenous fluids, and supportive care play a crucial role. However, depending on the degree of hypertriglyceridemia, the role of other treatment options may need to be implemented. There are no set established guidelines for the management of hypertriglyceridemia-induced pancreatitis, but the role of insulin, heparin, and plasmapheresis has been studied and successfully used in its management. We report the case a 44-year-old female with clinical acute pancreatitis secondary to hypertriglyceridemia who was successfully managed with the addition of intravenous insulin.

## Introduction

Acute pancreatitis is a life-threatening inflammatory condition of the pancreas which has a yearly incidence in the United States estimated to be about 40 cases per 100,000 adults [1]. In severe hypertriglyceridemia (triglycerides > 1,000 mg/dL) a rapid reduction of the triglyceride needs to be achieved. Hypertriglyceridemia is the most common cause for acute pancreatitis after gallstones and alcohol [2, 3]. In emergent cases apheretic treatment can be considered. After reviewing and analyzing current literature, apheresis is a useful therapeutic tool in its management; however, it has its limitations due to its availability and high costs. The standard treatment of hypertriglyceridemia involves medical management, for example with omega-3-fatty acids; however, in severe cases resulting in acute pancreatitis, insulin has also been successfully used for its management. Thus, this case report and review of the medical options of severe hypertriglyceridemia-induced pancreatitis will be demonstrated in order to better understand its treatment and awareness.

## Case Report

A 44-year-old Hispanic female with a past medical history only of hypertension presented with progressively worsening, generalized, non-radiating abdominal pain of 3 days duration associated with vomiting but not with food intake. As per the patient, her appetite had decreased markedly and there were no episodes of hematemesis or melena. Other significant portions of her history demonstrated that she had undergone cholecystectomy 5 years prior and she denied any alcohol use.

Upon physical examination, the patient was in moderate pain with a blood pressure of 112/74 mm Hg, pulse 81/min, respiration rate 16/min, saturation 99% on room air, and temperature of 99.9 °F. Abdominal examination revealed generalized tenderness in the epigastric region without rebound tenderness or guarding. Laboratory data showed a hemoglobin of 13.9g/dL, hematocrit 35%, WBC 11.1 × 10^3^/µL, platelets 286 × 10^6^/μL, glucose 136 mg/dL, BUN 8 mg/dL, creatinine 0.8 mg/dL, calcium 8.8 mg/dL, albumin 3.7 mmol/L, total protein 6.8, sodium 125 mmol/L, potassium 4.4 mmol/L, chloride 88 mmol/L, bicarbonate 2 mmol/L, lipase 145, alkaline phosphatase 69 U/L, total bilirubin 0.5 mg/dL, AST 47 U/L, and ALT 41 U/L.

**Figure 1:**
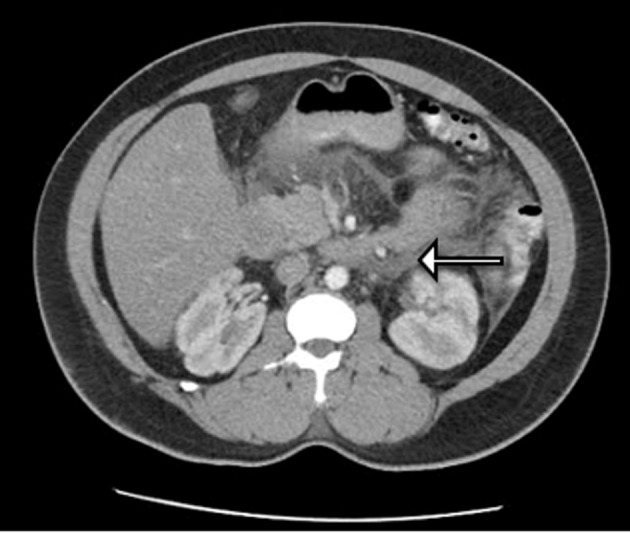
CT of abdomen demonstrating degree of pancreatitis.

Further analysis included computed tomography scan of the abdomen correlating with acute pancreatitis involving the head, uncinate process and pancreatic duodenum but no pancreatic ductal dilatation or obvious calcification (Fig. 1). Right upper quandrant ultrasound was also completed; however, it was found to be unremarkable.

**Figure 2:**
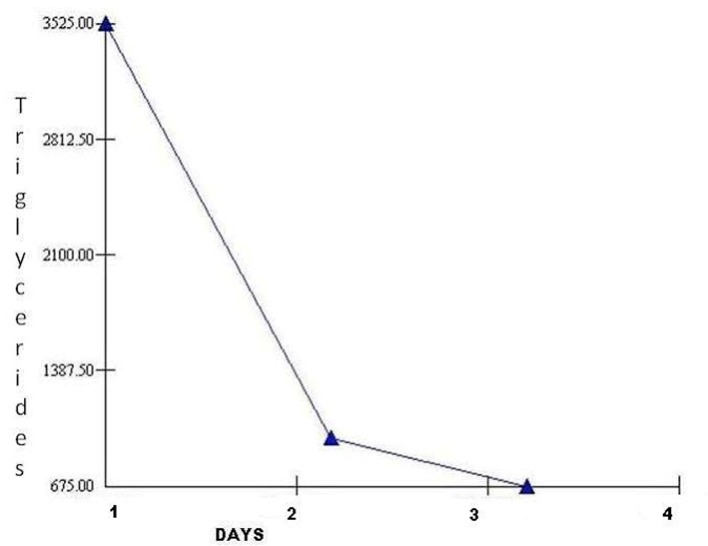
Triglyceride level vs. hospital stay.

The patient was admitted to the hospital with the diagnosis of acute pancreatitis, initially managed with bowel rest and supportive care which did not result in any improvement in her symptoms. Patient’s triglyceride level was then reported to be elevated at 3,525 mg/dL and she was transferred to intensive care unit and insulin drip was initiated. Patient’s triglyceride level decreased to 973 mg/dL the next day and the patient’s symptoms resolved (Fig. 2).

## Discussion

Hypertriglyceridemia may result in acute pancreatitis in up to 7% of cases; however, it rarely occurs except when triglycerides levels are greater than 1,000 mg/dL [2, 3]. It is also important to note that in some cases serum amylase levels may not be significantly elevated due to the interference from the triglycerides with certain amylase assays. A possible cause of this form of pancreatitis is thought to be the result of damage to acinar cells and microvascular membranes from elevated free fatty acid and lysolecithin formation in the pancreatic bed from lipoprotein substances [4]. The case discussed is also an example of a scenario which is an even rarer cause of acute pancreatitis induced by hypertriglyceridemia. It has been shown that non-diabetic, non-alcoholic, non-obese patients with hypertriglyceridemia which is diet or drug induced appear to account for only 15% of acute pancreatitis associated with hypertriglyceridemia [5].

Initial treatment is similar to any case of acute pancreatitis, which involves pancreatic rest by decreased oral intake, intravenous hydration, and pain management. However, after lack of clinical improvement and significantly elevated triglyceride levels, other options need to be considered. Although no standard guidelines are present for its management, insulin infusion is effective in decreasing triglyceride levels [6]. The mechanism of action behind this form of treatment suggests that insulin increases lipoprotein lipase (LPL) activity which can degrade chylomicrons and thus reduce serum triglycerides [7]. Another form of medical management which remains controversial is the use of heparin which can stimulate the release of endothelial LPL into circulation; however, it may only result in transient rise in LPL followed by increased degradation of plasma stores causing LPL deficiency [8].

In certain cases, a successful method of treatment relies on apheresis for the lowering of triglyceride levels. This was first reported in 1978 by Betteridge et al and can result in a rapid decrease in triglyceride levels over a short period of time compared to the other treatment options described [9]. Through the use of plasmapheresis compared to insulin use, a triglyceride level reduction of 65-70% has been documented [8]. This reduction was well studied and illustrated in a retrospective study of five cases by Lennertz et al managed with apheresis directly after diagnosis [10]. However, in the literature it has been shown that the timing and early use of apheresis is crucial in its benefit. Furthermore, due to its lack of availability at times, risks, and expense; insulin can be used as an alternative and safer method of treatment.

### Conclusion

Severe hypertriglyceridemia is a rare cause of acute pancreatitis especially in non-alcoholic, non-obese, non-diabetic patients and has a few options for its management. Even after supportive care, the lack of awareness of proper treatment modalities available can result in discomfort and prolonged hospital stays in patients presenting with such a diagnosis. Although, plasmapheresis is useful, it can be found to be an expensive treatment option and is not available in all medical centers. Thus, the use of insulin infusion as seen in this case report has been demonstrated to be a viable option for rapid improvement in symptoms associated with acute pancreatitis associated with elevated triglycerides.
